# A large European diversity panel reveals complex azole fungicide resistance gains of a major wheat pathogen

**DOI:** 10.1128/mbio.02766-25

**Published:** 2025-11-24

**Authors:** Guido Puccetti, Thomas Badet, Daniel Flückiger, Dominique Edel, Alice Feurtey, Camille Delude, Emile Gluck-Thaler, Stefano F. F. Torriani, Gabriel Scalliet, Daniel Croll

**Affiliations:** 1 Laboratory of Evolutionary Genetics, Institute of Biology, University of Neuchâtel 27214, Neuchâtel, Switzerland; 2 Syngenta Crop Protection AG 548990 , Stein, Switzerland; Universidad de Cordoba, Cordoba, Spain

**Keywords:** fungicide resistance, *Zymoseptoria tritici*, demethylation inhibitors, genome-wide association study, agriculture

## Abstract

Fungicide resistance in crop pathogens poses severe challenges to sustainable agriculture. Demethylation inhibitors (DMIs) are critical for controlling crop diseases but face rapid resistance gains. Even though the main molecular basis of resistance is well established, field surveys have repeatedly revealed alternative resistance mechanisms. The European continent has seen rapid and heterogeneous gains in azole resistance. Here, we establish a large genome panel to dissect the genetic architecture of emerging resistance in the major wheat pathogen *Zymoseptoria tritici*. The European diversity panel spans 15 sampling years and 27 countries for a total of 1,394 sequenced and phenotyped strains. Using two complementary assays to quantify resistance levels of each strain, we captured fine-grained shifts in DMI resistance over space and time. We conducted genome-wide association studies based on a comprehensive set of genotyping approaches for six DMIs. We mapped a vast array of loci encoding target functions, but also diverse channel, kinase, phosphotransferase, oxidoreductase, and monooxygenase functions. The substantial scope in genetic mechanisms underpinning DMI resistance significantly expands our mechanistic understanding of how continent-wide resistance arises in fungal pathogens over time. Diversification of the *Cyp51* coding sequence was particularly striking with new resistant haplotypes emerging with complex configurations and geographic patterns. Finally, we functionally assessed the impact of resistance-associated synonymous mutations in *Cyp51* in a sensitive and resistant background. However, no significant contribution was detectable, suggesting that associations were caused by linkage disequilibrium. This study provides expansive new insights into fungicide resistance gains of crop pathogens relevant for future resistance management strategies.

**IMPORTANCE:**

Sustainable food production requires the management of disease agents attacking crops. Application of antifungal compounds is among the key elements of pathogen containment; however, resistance can rise rapidly, leaving crop plants vulnerable to resurgent pathogen attacks. Most research has focused on establishing the molecular basis of fungicide resistance, studying mutant strains under laboratory conditions. How fungicide resistance is gained in agricultural fields over space and time remains poorly understood. Our work introduces the largest yet systematic collection of strains of a crop pathogen to assess how resistance mutations arose against a major class of fungicides. Tracking the fungal wheat pathogen, *Zymoseptoria tritici*, reveals heterogeneous gains in resistance over the past decades across the European continent. Using association mapping techniques and functional validation, we disentangled a complex set of mutations contributing to resistance. Unraveling the complexity of concurrent resistance mutation gains helps focus resistance management practices in agriculture.

## INTRODUCTION

The application of fungicides in agriculture is the primary containment strategy to reduce crop losses caused by fungal pathogens (1). However, resistance can rise rapidly in fields following intense application of fungicides (2, 3). The speed of resistance gains and the geographic scope remain difficult to predict (2, 4). The emergence of fungicide resistance highlights the challenges of convergent adaptation across geographic space (5). Even though the molecular basis of fungicide resistance may be well established for specific pathogens and fungicides, field surveys have repeatedly revealed alternative resistance mechanisms (6–8). Demethylation inhibitors (DMIs) are among the most widely applied fungicides (1). Resistance to this class of fungicides is mainly attributed to mutations in the gene encoding the molecular target *Cyp51*, which encodes the enzyme sterol 14α-demethylase (9, 10). This enzyme is essential for the ergosterol biosynthesis pathway underpinning the production of sterols for fungal cell membranes (10, 11). A number of recent studies showed that DMI resistance is also mediated by additional mechanisms beyond mutations in the *Cyp51* gene, such as regulatory changes in promoter elements leading to overexpression, as well as non-target resistance caused by the overexpression of efflux pumps (12–14). In crop pathogens, field resistance to DMIs has risen due to a combination of different on- and off-target mechanisms (7, 15). Off-target mechanisms, such as the combinatorial effects of multiple genetic factors, have been particularly significant. For instance, resistance to fluconazole in the human pathogen *Candida albicans* can be gained in complex steps mediated by overexpressed NCP1, cytochrome P450, and loss of heterozygosity at *KSR1* involved in sphingolipid biosynthesis in addition to chromosome copy-number variation (14). In agriculturally relevant pathogens, off-target fungicide resistance can be mediated by detoxification (7, 8, 16, 17), regulation of stress response pathways (18, 19), and mitochondrial functions (6, 20, 21). However, it remains largely unknown how such combinatorial effects of resistance mutations contribute to the gain of fungicide resistance over space and time in the field.

Population genomic surveys of pathogen populations have facilitated tracking fungicide resistance against DMIs and other fungicides at the field and continental scales. Recent gains in DMI resistance in the fungal wheat pathogen *Zymoseptoria tritici* were likely underpinned by both convergent and unique resistance adaptations (1, 2, 4, 5). Resistance to the DMI propiconazole was associated with mutations in a gene encoding an aspartate-histidine-histidine-cysteine (DHHC) palmitoyl transferase and was geographically restricted to fields in the USA (22). Geographic variation in fungicide resistance has also been observed in *Fusarium fujikuroi,* with differing sensitivity levels reported between countries (23, 24). Regional differences in the rise of resistance toward succinate dehydrogenase inhibitor (SDHI) fungicides were likely in part caused by variation in selection strength and geographic isolation (25–27). Key insights into population-level resistance mutations were gained using genome-wide association studies (GWAS) associating phenotypic trait variation (i.e., fungicide resistance) with molecular variation (28–30). Applications of GWAS in *Z. tritici*, the barley scald pathogen *Rhynchosporium commune* and *Cercospora beticola* causing *Cercospora* leaf spot revealed both major contributions of *Cyp51* mutations, but also previously unknown loci contributing to DMI resistance (22, 30–33). GWAS further revealed complex associations between *Cyp51* mutations and levels of resistance toward different DMI fungicides. In *C. beticola,* a synonymous single nucleotide polymorphism (SNP) was associated with tetraconazole resistance as well as a further synonymous SNP underpinning *Cyp51* expression variation (31). However, significant associations of synonymous variants could also be explained by high linkage disequilibrium (LD), especially following a selective sweep for resistance gains.

Assessing fungicide resistance experimentally is highly sensitive to the composition of the test medium, temperature, and availability of the fungicide in the medium. Resistance is commonly assessed in liquid culture medium over an array of fungicide doses (34–36). However, many filamentous fungi do not grow well in submerged cultures, as they show unusual morphologies, including dispersed hyphae or form pellets (37). In contrast, solid media assays typically require larger amounts of fungicides compared to liquid-based methods and are challenging to standardize (38). Further complicating fungicide resistance phenotyping efforts are inconsistencies in resistance assessed in liquid versus solid media. Discrepancies in responses were observed, e.g., for *Clarireedia jacksonii* growth in the presence of propiconazole in liquid and solid media (39). The reasons for the discrepancies remain unclear; however, non-linear relationships between growth responses in different media are a likely factor. Even though growth on culture medium may produce binary growth/no-growth responses facilitating the identification of resistant genotypes, colony growth responses to fungicides can also show features of a quantitative trait (40). Hence, high-throughput assessment of variation in resistance requires efforts in standardization and protocol development.

With the widespread application of DMIs on the European continent, fungal plant pathogens such as the wheat pathogen *Z. tritici* appeared as major threats given their ability to evolve resistance (41, 42). European wheat production heavily relies on DMIs for control to prevent yield losses on the order of 50% (43). *Z. tritici* populations show remarkable evolutionary potential to overcome host resistance and the application of fungicides. Adaptation is facilitated by high levels of genetic variation across both field and continental scales (29, 44), including structural variation and the presence of accessory chromosomes (45–47). Furthermore, high rates of recombination (46, 48) and the activity of transposable elements (TEs) contribute to highly diverse populations (49, 50). Gains in DMI resistance were in part mediated by numerous point mutations in the target gene *Cyp51* (25, 51–53). *Z. tritici* populations are characterized by a complex mixture of resistant *Cyp51* haplotypes underlying a differential effect of mutations across DMIs (52, 54, 55). Some key non-synonymous mutations include Ser524Thr, which increases resistance against at least four DMIs (51, 54, 55). The Ile381Val substitution underpins resistance against two additional DMIs (56). *Z. tritici* also evolved non-target site mechanisms to tolerate DMIs (7, 22, 57, 58). This includes the overexpression of the efflux pump encoded by *MFS1* in *Z. tritici* (7). Monitoring of multidrug resistance, including terbinafine, identified variation beyond the known *MFS1* promoter variants, suggesting that additional DMI non-target site resistance mechanisms remain to be discovered (57). The complexity in mutations contributing to DMI resistance raises significant questions about the geographic scope and convergence in resistance gains. Such a lack of knowledge also limits our ability to predict the emergence of resistance and protect crops effectively.

Here, we establish and investigate the largest hierarchically collected sampling of *Z. tritici* across the European continent covering a time span of 15 years. We optimized phenotyping assays to comprehensively capture shifts in DMI fungicide resistance across 1,394 strains. GWAS conducted for six DMIs identified resistance mechanisms associated with target functions, but also diverse channel, kinase, phosphotransferase, oxidoreductase, and monooxygenase functions. Resistance mutations were gained heterogeneously among the major wheat-producing areas. Finally, we assessed contributions of synonymous and non-synonymous coding sequence mutations in *Cyp51* and confirmed a resistance shift caused by a specific emerging *Cyp51* haplotype.

## RESULTS

### A European diversity panel to survey resistance emergence

**Fig 1:**
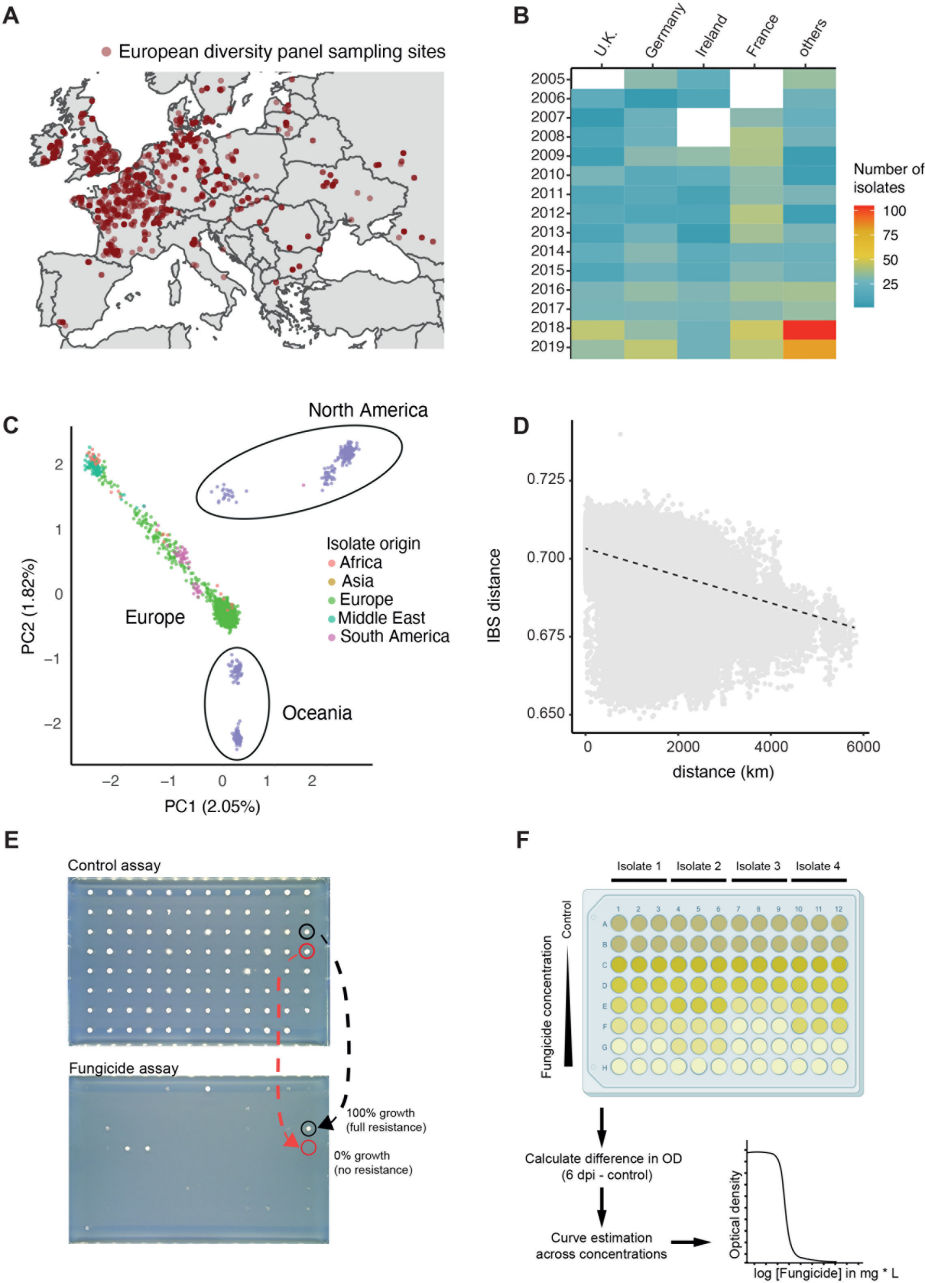
Collection and analyses of the fungal wheat pathogen *Zymoseptoria tritici* to create a European diversity panel (*n* = 1,394 strains). (**A**) Sampling distribution across Europe of the European diversity panel. (**B**) Heatmap representing the number of strains sampled in France, Germany, the UK, Ireland, and the rest of Europe. (**C**) Principal component analysis of 2,156 strains, including a separate strain collection covering additional continents. (**D**) Isolation-by-distance analyses using identity-by-state (IBS) pairwise distances. (**E**) Relative growth was determined by comparing the fungal growth in fungicide-treated medium to that in control medium after 7 days. The growth area for each strain in the fungicide condition is normalized to the control condition. Red circles indicate strains with 100% resistance, while black circles represent strains with 0% resistance. (**F**) Schematic representation of the estimation of half-maximal effective concentrations (EC_50_). Fungal strains were exposed to varying fungicide concentrations, and optical density (OD) measurements were taken at 0 days and 6 days post-inoculation (dpi). OD values are used to calculate EC_50_ values based on a non-linear logistic regression.

To investigate the genetic basis of DMI resistance under field conditions, we selected strains collected on infected wheat leaves across diverse regions in Europe. Sampling was particularly dense in Central and Northern European regions of intense wheat production and exposure to more fungicide applications (59). Strains originated from 27 countries sampled over a time span of 15 years (Fig. 1A) (see Table S1 at https://doi.org/10.5281/zenodo.17063534). For this, we subset a larger collection of *Z. tritici* strains assembled from fungicide resistance monitoring efforts across the continent. We prioritized strains using a hierarchical sampling approach, focusing first on selecting equal numbers of strains in 100 km^2^ areas defined across Europe. Within these areas, we favored strains from distinct wheat fields, at larger geographic distances, and spread over the available sampling years. We complemented the 100 km^2^ area sampling if more strains were available (see Materials and Methods for further details). This selection process maximized the covered geographic area and timespan. Strains from Germany, France, the UK, and Ireland accounted for ~72% of the panel (Fig. 1B).

Each *Z. tritici* strain of the European diversity panel was prepared for long-term preservation, phenotypic assays, and whole-genome Illumina sequencing. We followed a previously established genotyping and filtering procedure to obtain the most robust set of SNP and short indel variants (29). Even though all strains were sampled from European wheat fields, previous population genomic analyses showed that gene flow is ongoing, and this might lead to heterogeneous pools of genotypes across continents (29). Such heterogeneity could negatively impact the power of GWAS. Hence, we contextualized the genetic diversity of the European diversity panel with the known global genomic diversity of the species by exploring diversification in a joint analysis with the previously established thousand-genome panel (29). This resulted in a combination of 1,134 newly and 1,022 previously sequenced data sets for a total of 2,156 high-quality genomes. We identified 8,536,499 raw SNP and 6,606,877 raw indel candidate loci. After stringent quality filtering, we retained 472,041 SNPs for the European diversity panel, with minor allele frequencies of at least 5% for further processing. The global genetic diversity of the species was primarily structured into a North American, an Oceanian, and a dispersed third group, including strains originating from Europe, the Middle East, North Africa, and South America, consistent with previous genetic diversity analyses (29) (Fig. 1C). The European diversity panel showed a striking spread in genetic diversity, consistent with Europe being a founder population for other continents as well as experiencing recent incoming gene flow.

Focusing only on genomic data of the new European diversity panel, we identified fine-scale admixture patterns characterized by 11 genetic clusters (see Fig. S1A through C at https://doi.org/10.5281/zenodo.17063534). Gene flow has occurred over large distances, as indicated by a slow decay of identity-by-state (IBS) over geographic distance (Fig. 1D). Genetic differentiation among major wheat producers, i.e., Germany, France, the UK, and Ireland, was generally low, consistent with extensive gene flow across Europe (see Fig. S1D at https://doi.org/10.5281/zenodo.17063534). To assess mapping power in the European diversity panel, we investigated LD decay and found that *r*² decayed to ~0.2 within ~400 bp (see Fig. S1E at https://doi.org/10.5281/zenodo.17063534). Rapid LD decay is a hallmark of highly diverse populations with high effective population size.

### Profiling fungicide resistance across the continent

We assessed resistance in the strain panel against DMIs including prothioconazole, prochloraz, epoxiconazole, tebuconazole, and cyproconazole, which were all applied in Europe to control *Z. tritici* (see Table S2 at https://doi.org/10.5281/zenodo.17063534) (60). We first assessed geographic variation and temporal shifts in resistance levels using liquid culture assays to determine half-maximal effective concentrations (EC_50_). Resistance levels were significantly higher in strains from Western and Northern Europe (i.e., France, Ireland, and the UK) compared to the rest of the continent (see Fig. S2A at https://doi.org/10.5281/zenodo.17063534). From 2005 to 2019, resistance increased across most sampled regions, consistent with broad fungicide resistance gains on the continent (see Fig. S2B at https://doi.org/10.5281/zenodo.17063534). Overall, we determined EC_50_ values for the following fungicides and sample scopes: cyproconazole (*n* = 1,326), tebuconazole (*n* = 1,122), epoxiconazole (*n* = 1,061), prochloraz (*n* = 1,236), and prothioconazole (*n* = 1,324), with 967 isolates having EC_50_ measurements for all five fungicides. We complemented fungicide resistance EC_50_ with colony growth assessments on solid medium (with or without fungicide; Fig. 1E and F). This allowed us to determine binary growth/no-growth responses in the presence of the following fungicides: cyproconazole (*n* = 464), tebuconazole (*n* = 141), epoxiconazole (*n* = 464), prochloraz (*n* = 172), prothioconazole (*n* = 925), and mefentrifluconazole (*n* = 899) with 39 isolates having relative colony size measurements for all six fungicides. We investigated the degree of convergence in resistance assessed from colony growth on solid medium versus liquid culture EC_50_ values. For cyproconazole, 306 strains showed no growth on solid medium at 10 mg L⁻¹, yet liquid assays revealed EC_50_ values ranging from 0.018 to 71.05 mg L⁻¹, with 277 strains with EC_50_ values lower than 1 mg L⁻¹. Similarly, for prothioconazole, 311 strains showed no growth at 1 mg L⁻¹ on solid medium, while EC_50_ values in liquid assays spanned 0.007 to 72.0 mg L⁻¹, with 322 strains with EC_50_ values lower than 1 mg L⁻¹. The divergence highlights how resistance assays can influence the detection of cross-resistance patterns. The observed discrepancies highlight the relevance of assessing fungicide resistance in multiple morphological states spanning a range of concentrations.

### Complex genetic architecture of resistance to DMIs

**Fig 2:**
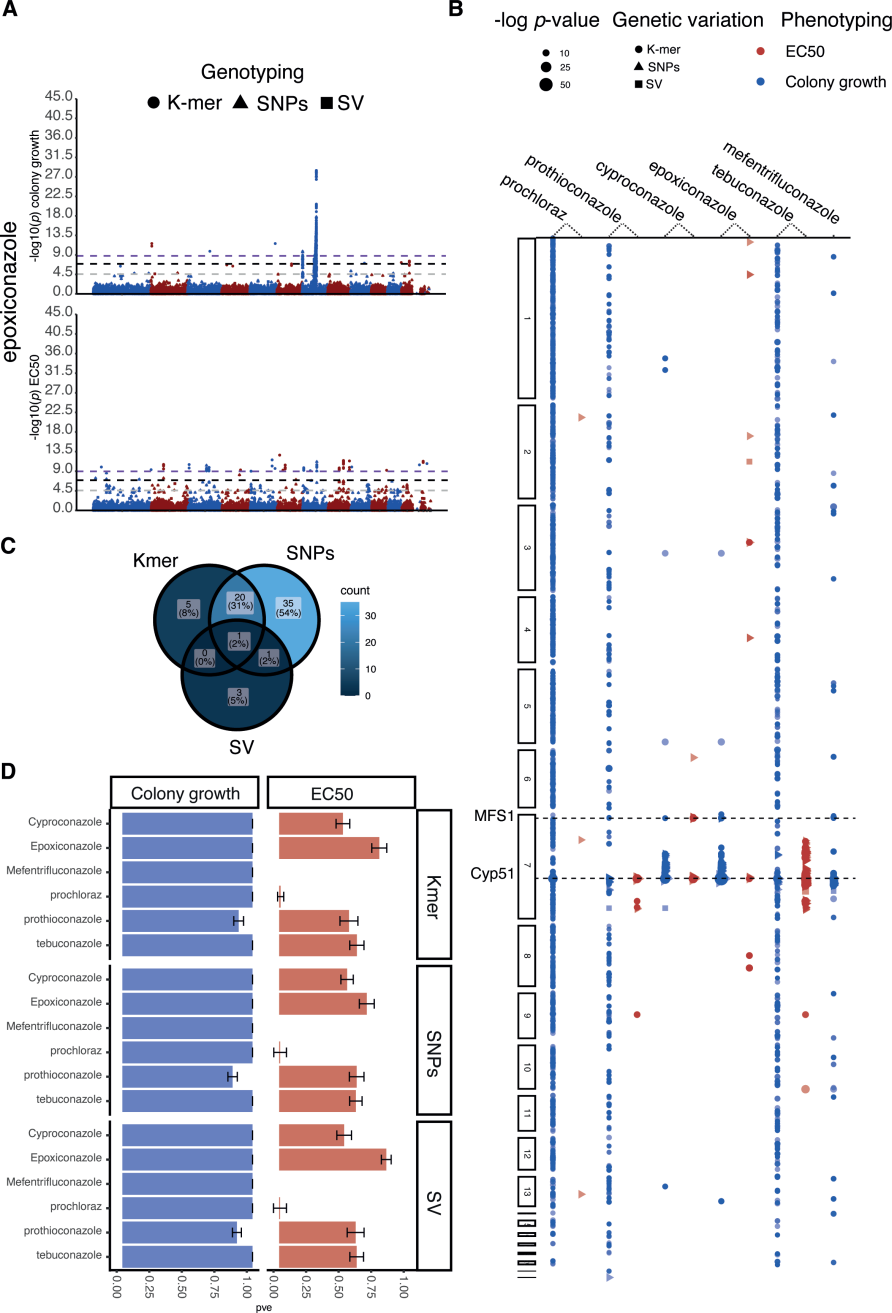
Genetic architecture of DMI resistance detected across the European diversity panel. (**A**) Manhattan plot of epoxiconazole resistance based on relative growth values (top) and EC_50_ estimates (bottom). For *P-*values (Wald) >0.0001, we retained only 1 out of 30 positions for visualization purposes. (**B**) Scatter plot of *P-*values (Wald) crossing the Bonferroni threshold. The *y*-axis represents coordinate-ordered genes split by chromosome with a significantly associated SNP for variation in resistance to six DMIs (relative growth and EC_50_). The shape of the variants corresponds to SNPs (dots), k-mers (triangles), and structural variation (square). For additional information and gene identifiers, see Supplementary S7. (**C**) Venn diagram of gene numbers with significant associations for either SNPs, k-mers, and structural variants (SVs) based on EC_50_ estimates. The overlaps define genes identified based on multiple genotyping approaches. (**D**) Box plot of the chip heritability for SNPs, k-mers, and SVs across the six DMIs tested based on relative growth and EC_50_ values.

We mapped mutations underpinning DMI resistance using GWAS. The DMIs prothioconazole, cyproconazole, prochloraz, tebuconazole, and epoxiconazole were assayed both in liquid (EC_50_) and on solid medium (Fig. 2A). We included solid media growth assay data for mefentrifluconazole, which is a DMI introduced only in 2019 to complement the panel of previously released DMIs. Mefentrifluconazole was reported to have high efficacy even on resistant *Z. tritici* populations (61). We investigated the relative contributions of different polymorphism classes by performing separate GWAS for three different genotyping data sets: (i) SNPs, (ii) large structural variation (>30 bp), which includes insertions, deletions, and complex regions, and (iii) 25 bp k-mer sequences derived directly from the short-read data sets in a reference-free approach. Significantly associated k-mers were then mapped to the reference genome, and associations are reported if a uniquely matching position was discovered. Furthermore, we evaluated binary colony growth data on solid medium versus colony sizes assessed as a quantitative metric of relative growth in the presence of fungicides versus the control plate (see Fig. S5A at https://doi.org/10.5281/zenodo.17063534). We repeated GWAS for the large prothioconazole data set using the different phenotype classification systems (binary vs continuous encoding). We found that 6 SNPs were significantly associated only in GWAS performed on the binary growth/no-growth trait data set, 33 SNPs only with the quantitative relative growth encoding, and 18 SNPs with both (see Fig. S4A and B at https://doi.org/10.5281/zenodo.17063534). These findings suggest that the continuous trait encoding may provide greater resolution and statistical power for detecting genotype-phenotype associations. However, given the single biological replicate available for growth assessments, we conservatively considered only EC_50_ and colony growth/no-growth values for further analyses.

We identified a total of 53,226 genetic variants associated with DMI resistance, of which 28,776 (54%) overlapped with protein-coding regions across 1,199 genes (Fig. 2B). The target gene *Cyp51* was universally mapped through associations with variation in resistance to all tested fungicides. Genetic variants near the multidrug resistance factor *MFS1* were associated with resistance to mefentrifluconazole, cyproconazole, prothioconazole, and epoxiconazole (Fig. 2B). Discovery of genetic variation associated with DMI resistance was dependent on the phenotyping assay, with 8,145 significant associations for EC_50_-based assays and 45,081 significant associations for colony growth assays (see Table S3 at https://doi.org/10.5281/zenodo.17063534). For epoxiconazole resistance, we detected 297 significant k-mers, 10 SNPs, and one structural variant based on the EC_50_ assays, whereas colony growth assays revealed 7,843 k-mers, 161 SNPs, and three structural variants (Fig. 2A). This discrepancy of associated variants may reflect differences in assay sensitivity and dose-response properties to different DMI fungicides. The pattern of broad-spectrum associations (i.e., associations across many different gene functions) is likely driven by several factors, including the fact that some fungicides (tebuconazole, prochloraz, and prothioconazole) revealed many weakly associated loci rather than major locus effects. Furthermore, some associations are the likely result of linkage disequilibrium. Despite this potential for background noise in the overall associations, a number of individual genes stood out as highly significant, most notably a P450 gene (Zt09_7_00450, *P* = 2.49e − 62) and several major facilitator superfamily (MFS) transporter genes (Zt09_10_00549, *P*-values (Wald) = 5.57e − 19; Zt09_7_00012, *P* = 9.97e − 13; Zt09_7_00453, p-values (Wald) = 1.69e − 18).

Next, we investigated loci depending on whether these were associated with resistance to one or multiple fungicides. Focusing on EC_50_ measurements, we found 17 SVs, 52,157 k-mers, and 1,052 SNPs significantly associated with mefentrifluconazole, epoxiconazole, prochloraz, cyproconazole, and tebuconazole resistance. Among the SVs, we found insertion/deletions spanning 35 bp–221 bp and complex regions carrying non-homologous sequences of 751 bp–1,151 bp. An SV located 1,387 bp downstream of *Cyp51* was associated with mefentrifluconazole, epoxiconazole, tebuconazole, and cyproconazole resistance. Epoxiconazole resistance revealed three significant SV associations and 7,843 k-mer associations for colony growth, whereas associations with tebuconazole in EC_50_-based assays and mefentrifluconazole on solid medium revealed substantial diversity, with 6,720 and 8,867 significant k-mers identified, respectively, compared to only 108 and 560 associated SNPs (see Fig. S6 at https://doi.org/10.5281/zenodo.17063534). Resistance to prochloraz included two significantly associated SVs in any of the two assay types (Bonferroni threshold, α = 0.05), but 7,541 significantly associated k-mers on solid medium and 18 k-mers and three SNPs based on EC_50_ measurements (see Table S3 at https://doi.org/10.5281/zenodo.17063534). Variation in the types of associated loci for the same assay may stem from challenges in robustly genotyping all genetic variants. However, variation in associated loci between assay types for the same DMI is most likely explained by distinct genetic contributions to colony growth or growth in liquid medium under fungicide stress.

For EC_50_ assays, we found that 8% (5/65) of the genes were uniquely associated through k-mer genotyping, 5% (3/65) of the genes were uniquely associated with SV variants, and 54% (35/65) were uniquely associated with SNPs (Fig. 2C). A total of 31% (20/65) of the genes were identified both by SNP and k-mer-based associations, with only 2% (1/65) of the genes being associated with all three genotyping methods. The genes identified included only the gene Zt09_7_00444, of unknown function. Finally, we found 117 genes with significant associations for multiple fungicides or genes with significant associations for both phenotyping assays. To quantify how alternative genotyping strategies based on SNPs, k-mers, or SVs facilitate GWAS discovery, we estimated the heritability of associated variants for each DMI and assay type. We found that SV-based associations explained, overall, a higher proportion of variation in resistance, followed by k-mers and SNPs, suggesting that k-mer variants capture more comprehensively genetic variation underlying resistance (Fig. 2D). The heritability estimates for k-mer associations among DMIs and phenotyping assays ranged from 0.01 to 0.99, with an average of 0.98 for solid medium and 0.48 for EC_50_ measurements. SNP-based associations explained heritability estimates of 0.974 for solid medium and 0.477 for EC_50_. SV-based associations explained heritability of 0.980 for solid medium and 0.52 for EC_50_ (Fig. 2D).

### Gene functions associated with DMI resistance

**Fig 3:**
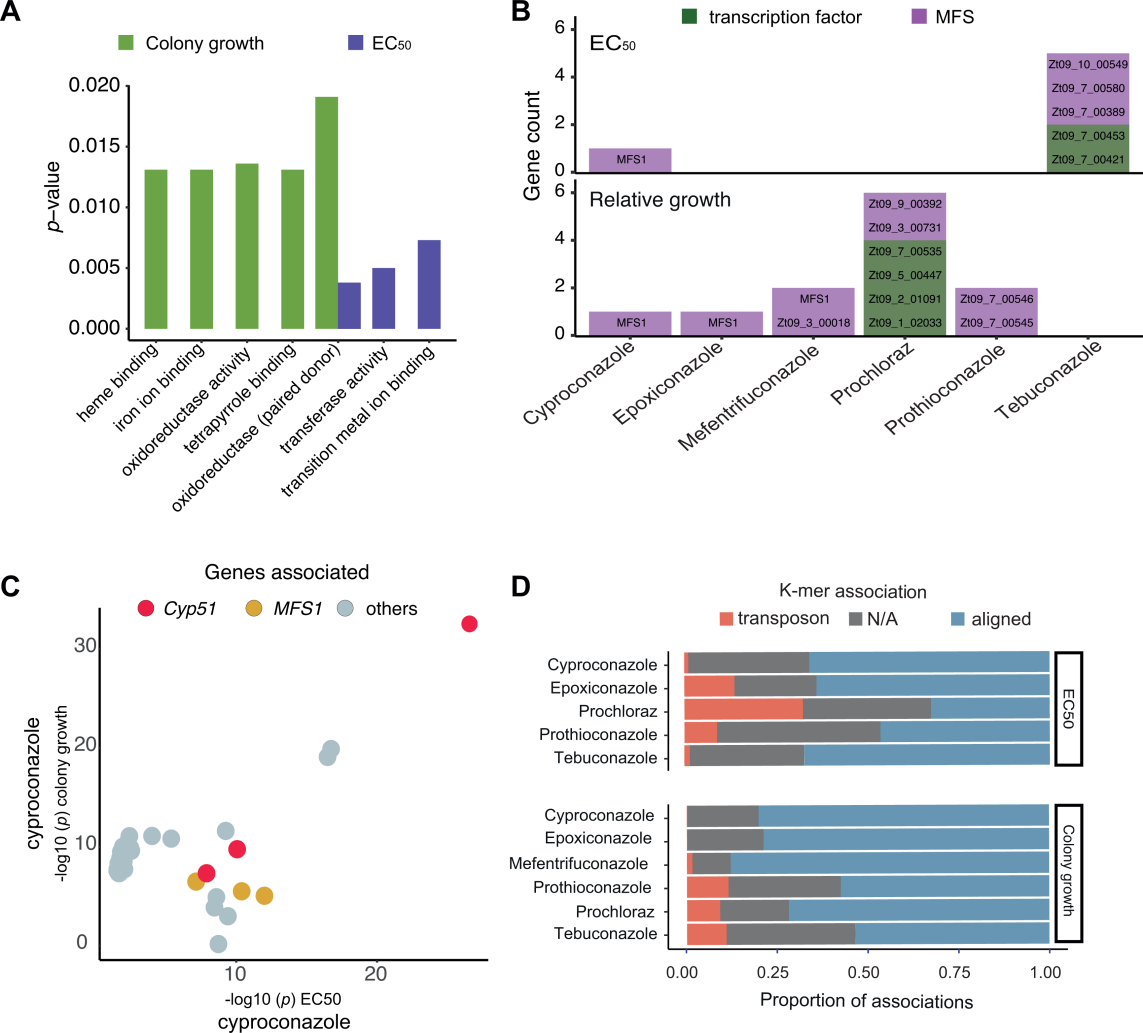
Transcription factors and MFS as resistance factors against DMIs. (**A**) Enrichment for gene ontology (GO) terms. MFS and transcription factors were identified according to ontology terms for MFS (GO:0055085–GO:0016021) and transcription factor (GO:0006355), respectively. (**B**) Enrichment analysis of the gene functions identified through GWAS based on EC_50_ and relative growth. Gene names are as follows: in green transcription factors (Zt09_TU_chr_1_02033, ...2_01091, ...5_00447, ...7_00535, ...7_00421, ...7_00453), in purple MFS (Zt09_TU_chr_10_00549, ...3_00018, ...3_00731, ...7_00389, ...7_00545, ...7_00546, ...7_00580, ...9_00392), as well as *MFS1* (Zt09_TU_chr_7_00012). Gene annotations follow Grandaubert et al. (66). (**C**) Visualization of *P*-values for the same SNPs based on GWAS using either relative growth or EC_50_-based resistance phenotyping. (**D**) Percentage of k-mers aligning to the reference genome (red), to TEs (blue), and not aligning (gray).

We identified a broad range of molecular functions contributing to DMI resistance. We performed an enrichment analysis on associated gene functions and found heme, iron ion, and tetrapyrrole binding, as well as oxidoreductase activity, enriched for solid medium growth assays (Fig. 3A). In contrast, gene functions discovered through liquid EC_50_ assays identified functions associated with transition metal ion binding, transferase activity, and oxidoreductase activity, with the latter being consistently enriched independent of the assay type (see Fig. S3 at https://doi.org/10.5281/zenodo.17063534). Resistance to DMIs in *Z. tritici* and other filamentous fungi has been associated with efflux pumps (i.e., *MFS1*) (7, 62) and various transcription factors (17, 63, 64). Our analysis identified nine *MFS* genes based on results from colony growth (Zt09_TU_chr_3_00018, ...3_00731, ...7_00545, ...7_00546, and ...9_00392) and EC_50_ assays (Zt09_TU_chr_10_00549, ...7_00389, and ...7_00580), and with *MFS1* being associated in both conditions (see Fig. S7 at https://doi.org/10.5281/zenodo.17063534). MFS transporters are capable of exporting a diversity of compounds through cell membranes (8, 65). Notably, our GWAS identified that associations in *MFS* loci are consistent with cross-resistance levels described in previous studies investigating MFS functions (58). Transcription factors were associated with resistance variation for two fungicides (Fig. 3B). For prochloraz resistance, four transcription factors (Zt09_TU_chr_1_02033, ...2_01091, ...5_00447, and ...7_00535) contributed significantly to phenotypic variation, while two transcription factors (Zt09_TU_chr_7_00421, and ...7_00453) were associated with resistance to tebuconazole.

Finally, we compared the strength of SNP-based associations with DMI resistance across the two phenotyping methods, i.e., EC_50_ values and binary colony growth assessment. Our analysis revealed that the most significant associations varied depending on the phenotyping method, with some variants showing strong associations with either EC_50_ or solid medium growth but not in both assay types. Notably, SNP associations in the *Cyp51* gene were significant in both assay types at the Bonferroni threshold, indicating that *Cyp51* variants contribute substantially to DMI resistance regardless of the growth conditions (Fig. 3C). In conjunction, we demonstrate that DMI resistance across Europe has arisen from a combination of target site mutations in *Cyp51* as well as a wide range of non-target site mechanisms. Across all tested fungicides, *Cyp51* consistently appeared as the key genetic locus linked to resistance, highlighting the central role of this gene encoding the molecular target of DMI fungicides. Additional notable contributions are from transposable element polymorphism, highlighting the complex genetic landscape of DMI resistance in Europe (Fig. 3D).

### *Cyp51*-driven resistance gains

**Fig 4:**
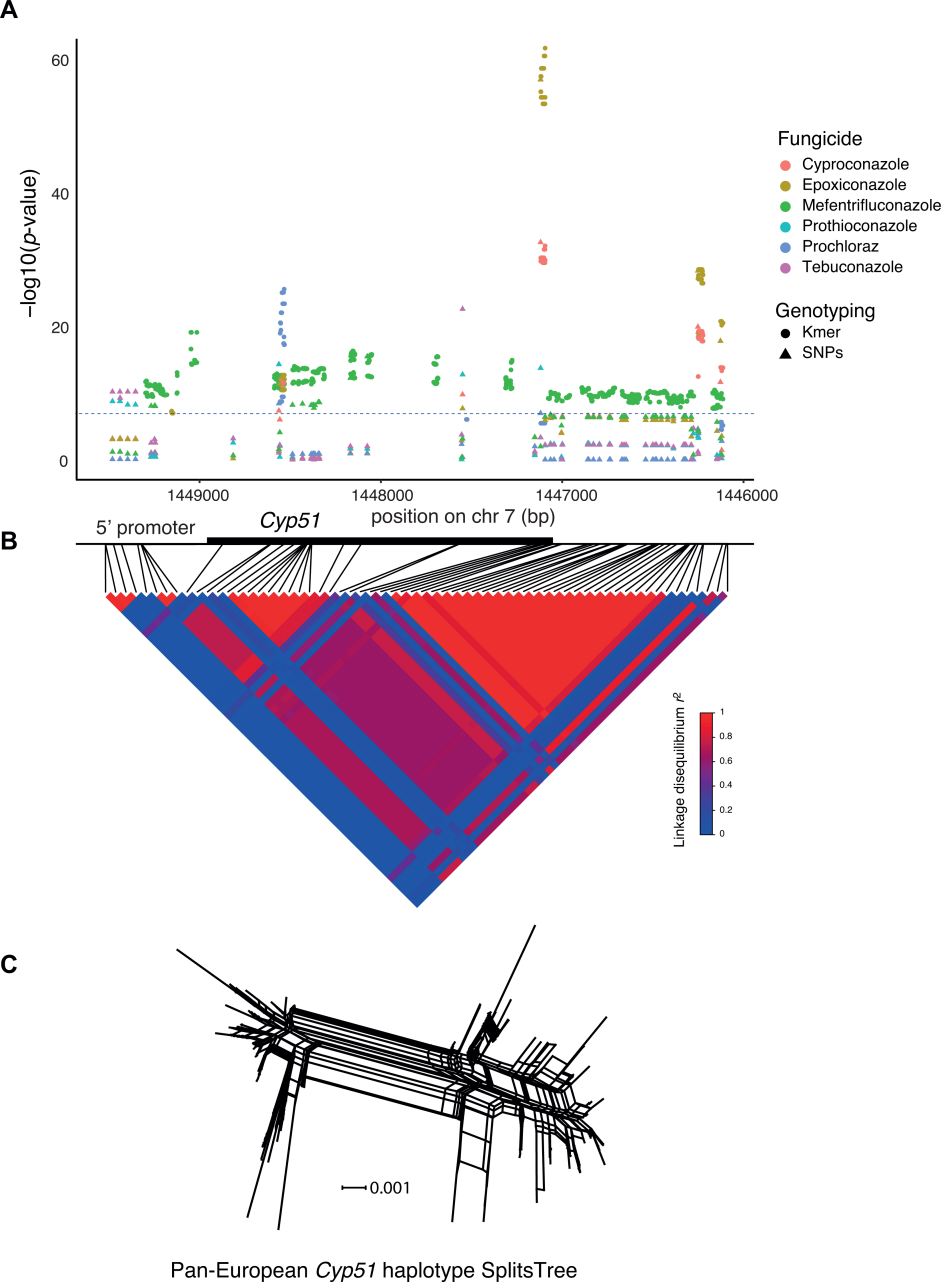
Complexity of *Cyp51* locus associations by SNP and k-mer variants. (**A**) Manhattan plot of SNPs (dots) and k-mers (triangle) associated with resistance to six DMIs. The black horizontal line shows the average SNP Bonferroni threshold (−log10 = 6.9) across the tested DMIs. (**B**) The coordinates show positions in *Cyp51* and the linkage disequilibrium heatmap. (**C**) Pan-European phylogenetic network of the *Cyp51* gene haplotypes constructed using SplitsTree.

We further examined the contributions of *Cyp51* alleles to DMI resistance across Europe, focusing on solid media growth (Fig. 4A and B). We identified 15 significantly associated SNPs, with 14 located in the coding sequence (CDS) and one in the intron for colony growth. Our GWAS confirm associations of previously documented missense resistance mutations (55, 67). For instance, the Ser524Thr variant showed an association *P* = 10^−55^ for epoxiconazole, *P* = 10^−32^ for cyproconazole, and *P* = 10^−13^ for prothioconazole. The Val136Phe mutation showed a *P* = 10^−11^ for epoxiconazole. Additionally, 904 significant k-mers overlapped the *Cyp51* CDS.

GWAS identified several synonymous variants within *Cyp51* associated with resistance. While most synonymous mutations were detected through SNP and k-mer analyses, such as Ser158Ser, Phe174Phe, Arg177Arg, Gly189Gly, Pro246Pro, and His277His, we identified additional synonymous substitutions, including Pro125Pro, Thr190Thr, and Leu327Leu, uniquely based on k-mer GWAS, suggesting that read alignment challenges against the reference genome might have prevented the discovery of all relevant SNPs in *Cyp51* (see Table S3 at https://doi.org/10.5281/zenodo.17063534). None of these synonymous variants were previously reported to be associated with fungicide resistance. Upon inspection, we found that some SNP-based genotyping failed to reveal these variants due to low SNP quality scores rather than a complete absence of evidence (see Table S4 at https://doi.org/10.5281/zenodo.17063534). None of these synonymous variants were previously reported to be associated with fungicide resistance. SNP-based genotyping was unable to identify most of these associations due to the stringent quality filtering applied to SNP variants (see Table S4 at https://doi.org/10.5281/zenodo.17063534).

To better understand the convergence in predicted effects of *Cyp51* mutations on DMI resistance, we investigated complements of missense and synonymous mutations for each DMI. We found that each DMI presented a unique combination of significantly associated variants. The smallest overlap in missense resistance variants was for tebuconazole against other DMIs, with only the Ile381Val mutation being shared. In contrast, the missense mutation Ser524Thr was associated with prothioconazole, cyproconazole, prochloraz, and epoxiconazole resistance. We also determined the resistance for 44 haplotypes representing the combinations of the most frequent 16 synonymous and missense mutations associated with DMI resistance (see Table S5 at https://doi.org/10.5281/zenodo.17063534). The large number of synonymous and missense mutations in *Cyp51* underpins a highly diverse set of haplotypes across the European continent. This is illustrated by the complex phylogenetic network of *Cyp51* haplotypes (Fig. 4C). As expected for the highly recombining populations of *Z. tritici, Cyp51* haplotypes show substantial diversification and significant evidence for recombination (*P* < 0.0001).

### Effects of *Cyp51* haplotypes on DMI resistance

**Fig 5:**
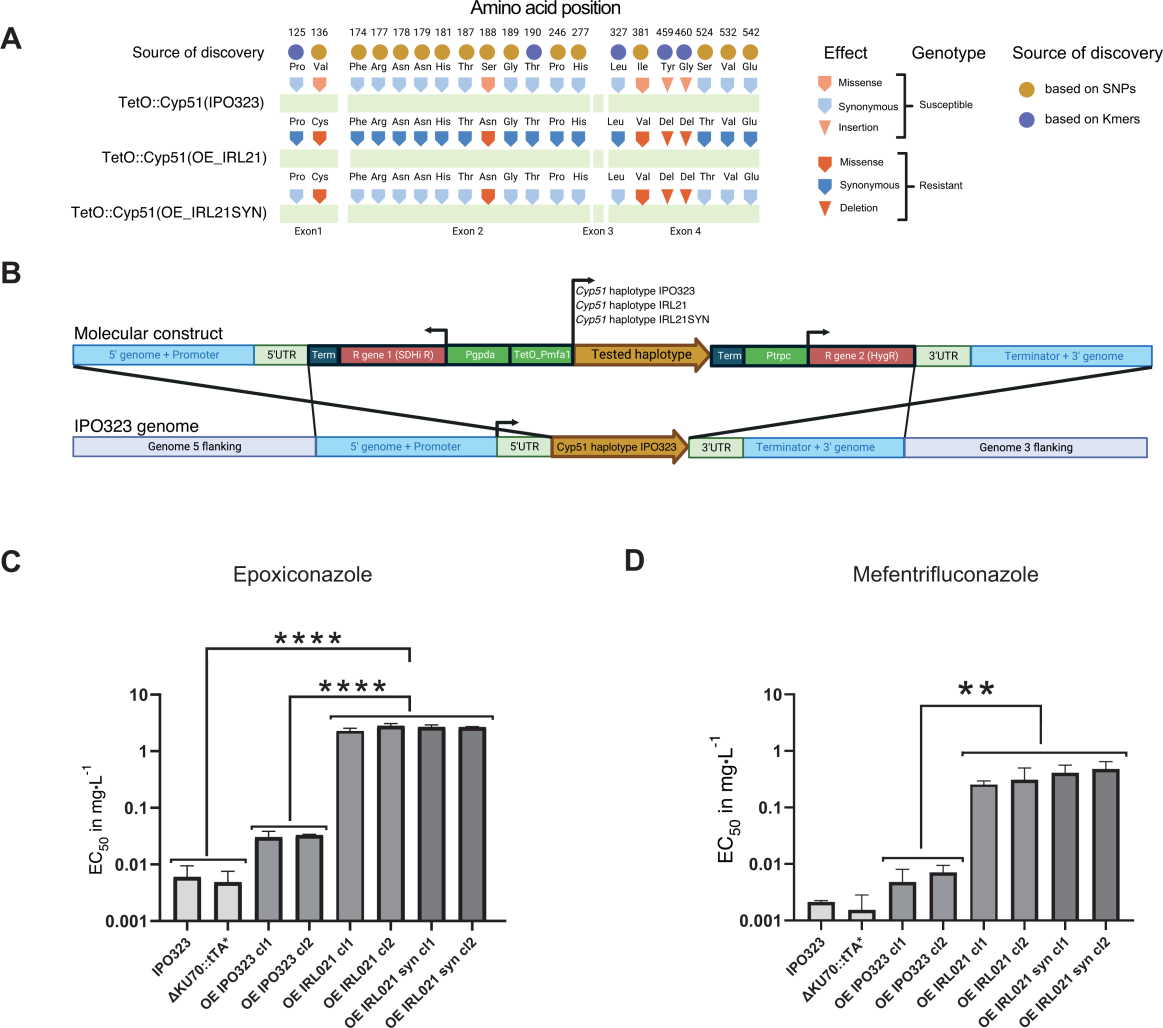
Molecular genetics analyses of *Cyp51* haplotype effects on DMI resistance. (**A**) Schematic representation of three different *Cyp51* haplotypes analyzed using allele swap experiments. These include the susceptible haplotype of IPO323 (wild-type, top row), the Irish strain 15STIRL21.1 (OE_IRL21, middle row), and synonymous variants only of the 15STIRL21 strain (OE_IRL21SYN, bottom row). All variants include a tetracycline-repressible promoter (TetO). The amino acid positions show the residues used to construct the mutants. Source of discovery refers to whether the amino acid position was found to be significantly associated with resistance either based on SNP or k-mer genotyping. Light residues match the haplotype of the susceptible genotype (wild-type IPO323), and dark residues match the resistant haplotype (15STIRL21 wild-type). Shapes used to represent residues highlight the predicted effect (i.e*.,* missense, synonymous, insertion/deletion; see figure for further details). (**B**) Construct diagram of the *Cyp51* haplotype swapping in the IPO323 ΔKU70::tTA* mutant background. The middle section of the construct carries one of the haplotypes of *Cyp51* (light brown) to be tested in the allele swap experiment, flanked by a tetracycline-repressible promoter (TetO_Pmfa1) in 5′ and by a terminator in 3′. This *Cyp51* expression cassette is itself flanked by two fungicide resistance cassettes (shown in red): isofetamid resistance and hygromycin resistance to exclude incomplete homologous recombination events during strain selection. Finally, 5′ and 3′ *Cyp51* flanking regions of the IPO323 genome were included for *in locus* homologous recombination. (**C**) Bar chart representing liquid culture EC_50_ values of the strains IPO323, ΔKU70::tTA*, OE_IPO323 (overexpression of IPO323 haplotype), OE_IRL21 (overexpression of IPO323 haplotype), and OE_IRL21SYN with epoxiconazole and (**D**) mefentrifluconazole. One-way analysis of variance: **** *P* < 0.0001, **P < 0.05.

Missense mutations in *Cyp51* are known to significantly affect DMI resistance. Here, we tested for effects of a haplotype carrying both significantly associated missense mutations as well as additional synonymous mutations. Using allele swapping, we formally tested for the contribution of either sets of missense or synonymous mutations on DMI resistance (see Table S6 at https://doi.org/10.5281/zenodo.17063534). We selected the *Cyp*51 haplotype of strain 15STIRL021.1 collected in Ireland in 2015. Strains carrying this haplotype display strong resistance toward DMIs and carry a number of resistance mutations (see Table S1 and S5 at https://doi.org/10.5281/zenodo.17063534; haplotype number 40). The *Cyp51* haplotype of 15STIRL021.1 includes four missense mutations (Val136Cys, Ser188Asn, Ile381Val, Ser524Thr) and an indel resulting in the deletion of two amino acids (Tyr459-Gly460Δ). To determine the impact of *Cyp*51 haplotypes experimentally, we swapped the wild-type copy of the IPO323 *Cyp*51 haplotype with either one of the three following haplotypes, each under the control of the same tetracycline-repressible promoter: (i) the 15STIRL021.1 *Cyp51* haplotype including all synonymous and missense mutations (OE_IRL21), (ii) the 15STIRL021.1 *Cyp*51 haplotype carrying all non-synonymous mutations but conserving original IPO323 codons for the 14 resistance-associated synonymous SNPs (OE_IRL21SYN), and (iii) a reintegration of the IPO323 haplotype as a control (OE_IPO323) (Fig. 5A).

We assessed DMI resistance of the *Cyp*51-swapped IPO323 mutants with epoxiconazole and mefentrifluconazole (Fig. 5B and C). On epoxiconazole, we detected an 80-fold difference in sensitivity between the control carrying the *Cyp*51 IPO323 haplotype and its counterpart carrying the 15STIRL021.1 haplotype (average EC_50_ of 0.031 versus 2.539 mg L^−1^, respectively), confirming a strong effect of this resistant haplotype. However, no significant difference was observed between the 15STIRL021.1 haplotype and its synthetic counterpart devoid of synonymous mutations (average EC_50_ of 2.539 mg L^−1^ and 2.673 mg L^−1^, respectively). We observed a similar resistance profile with mefentrifluconazole, with a 47-fold sensitivity difference between the control carrying the *Cyp*51 IPO323 haplotype and its counterpart carrying the 15STIRL021.1 haplotype (average EC_50_ of 0.005 mg L^−1^ versus 0.281 mg L^−1^, respectively) and no consistent differences between 15STIRL021.1 and its synthetic analog (average EC_50_ of 0.281 mg L^−1^ and 0.446 mg L^−1^, respectively; Table S7 at https://doi.org/10.5281/zenodo.17063534). These results suggest that synonymous SNPs of *Cyp51* do not significantly contribute to resistance. The IPO323 control mutant showed 3.88- and 6.52-fold EC_50_ difference compared to the background ∆KU70::tTA* (EC_50_ of 0.031 and 0.005, respectively) in epoxiconazole and mefentrifluconazole (analysis of variance [ANOVA]; *P* > 0.05). This could be explained by the weak overexpression of the target under the control of the Tet promoter. Since a slight transcriptional overexpression of *Cyp*51 might mask a weak effect of synonymous mutations on protein translation efficiency, we tested the sensitivity of the mutants downregulated for *Cyp51* expression. As expected, transcriptional repression of the molecular target with doxycycline sensitized all mutants carrying the gene under transcriptional control of the Tet promoter. The mutants carrying the IPO323 wild-type haplotype were strongly inhibited at the lowest epoxiconazole concentration in the test (0.00038 mg L^−1^), and the mutant carrying the 15STIRL021.1 haplotype and its synthetic counterpart showed approximately 15-fold higher sensitivity to epoxiconazole in the presence of doxycycline (EC_50_ of 0.173 and 2.539 mg L^−1^ for 15STIRL021.1 haplotype, respectively). However, the addition of doxycycline did not modify the lack of sensitivity differences between the 15STIRL021.1 haplotype and its synthetic counterpart lacking synonymous mutations (EC50 of 0.173 and 0.266 mg L^−1^, respectively, Fig. S8A at https://doi.org/10.5281/zenodo.17063534). As a control, we used benzovindiflupyr (an SDHI) and observed no significant differences among the IPO323 mutant strains (see Fig. S8B and C at https://doi.org/10.5281/zenodo.17063534). Hence, the assay did establish strong effects of the 15STIRL021.1 *Cyp51* haplotype compared to a sensitive strain. Even though synonymous mutations showed strong associations in GWAS, our assay confirmed only contributions by missense mutations to DMI resistance.

## DISCUSSION

We established a large strain panel for the major wheat pathogen *Z. tritici* to capture recent gains in fungicide resistance. The European diversity panel covers distinct agricultural regions with variable intensities of recent DMI fungicide application. Our genome sequencing analyses revealed that Europe harbors a highly diverse set of genotypes central to our understanding of *Z. tritici* diversity globally. We assessed two complementary sensitivity screening methods, revealing notable variation in resistance profiles across time and geography. We detected a total of 53,225 variants in 1,199 genes associated with resistance to different DMIs. The scope of the associations across the genome allowed for fine-grained analyses of cross-resistance among the structurally related DMIs, as well as to unravel patterns of parallel resistance evolution across the European continent.

The European diversity panel revealed extensive admixture across the continent, with only weak genetic differentiation (i.e., isolation-by-distance) over large distances. Exceptions included well-differentiated genetic groups sampled in Southern Europe (i.e., Italy, Spain, and southern France) and Eastern Europe (i.e., Russia), which likely reflects recent gene flow from North Africa and Central Asia, respectively (29). The substantial genetic diversity and weak differentiation are expected to empower GWAS by reducing spurious marker associations (68). Similarly, the resolution of GWAS is improved by reproducible phenotyping methods capturing traits relevant for resistance gains in the field. Leaf pathogens such as *Z. tritici* are usually exposed to fungicides during early disease development on the host. Fungicides act on the spore germination stage or at later mycelial development stages prior to full infection (69, 70). DMIs are known to inhibit fungal mycelial development stages (71). To maximize the discovery of potential resistance factors regardless of the morphological state of the strains, we performed both liquid culture and colony growth measurements. For most DMIs, colony growth in the presence of fungicides compared to control medium showed a largely bimodal distribution, and we proceeded with using these data as a binary growth/no-growth trait representing a conservative assessment of resistant versus susceptible genotypes.

Even though reproducibility of each resistance assay was high, resistance captured by each of the two phenotyping methods was only weakly correlated. This suggests that the pathogen expresses DMI resistance through at least partially distinct mechanisms in these two conditions. Furthermore, differences in the bioavailability of fungicides in liquid cultures compared to solid agar may explain additional discrepancies. For example, reduced oxygen levels in liquid culture and other microenvironmental factors may impact variation in the expression of resistance. The largest discrepancies between assays were found for prochloraz and prothioconazole. Loci mapping power may have been weakly affected by differences in sample size for the different fungicide assays. In addition, cryptic switches in growth morphology (yeast or filamentous growth) may also account for some of the observed discrepancies. As fungicide applications in the field typically occur on leaf surfaces and under aerated conditions, solid medium assays may better approximate such *in planta* exposure; yet fungal growth does not occur as a mycelial mass on the plant but rather spreads superficially before attempting to enter stomata. In contrast, liquid assays are highly reproducible and homogeneously expose fungal structures. However, some pleiotropic and stress-induced resistance mechanisms may not be observable in liquids. Discrepancies between exposure types have also been reported for *Cl. jacksonii* exposed to propiconazole, where liquid-based microplate assays detected resistant isolates missed by solid agar tests. This highlights that complementary assays, if feasible, can augment the spectrum of detectable resistance mechanisms through association mapping (39). The biological relevance of different assays needs to be carefully evaluated, though.

### Genetic architecture of DMI resistance across Europe

We identified a total of 53,226 genetic variants associated with DMI resistance, of which 28,776 (55%) were in protein-coding regions covering 1,199 distinct genes. Recent work revealed that a significant portion of DMI resistance is encoded by structural variation beyond SNPs (7, 22, 58). To map such genetic variations beyond point mutations, we implemented genotyping approaches suitable for the robust discovery of complex variants. We controlled for spurious genotypes by integrating reference genome-based controls for variants assessed from short-read data. Structural variants were identified based on a pangenome graph approach, and subread associations (i.e., k-mers) were cross-checked with reference genome mapping. For EC_50_ assessments, we found a single gene mapped by the three different genotyping approaches. K-mer- and SNP-based approaches revealed a shared gene set of 31% of the total. Among the enriched functional categories, several stand out as potentially relevant to fungicide resistance. These include ion transport and channel activity, which may influence drug uptake, efflux, and stress responses (58, 72). Kinase, phosphotransferase, oxidoreductase, and monooxygenase activity could contribute to fungicide metabolism, signaling pathways, and stress adaptation.

The gene encoding the DMI target *Cyp51* was well-resolved by GWAS based on EC_50_ and colony growth data, with 18 SNPs and 1,475 k-mers mapped to the coding sequence, further confirming that mutations in the target are the primary driver of DMI resistance in the field (51, 52, 55, 56). Similarly, the gene encoding the detoxification factor *MFS1* was mapped by 203 variants (6 SNPs and 197 k-mers). Previous work established that multidrug resistance by *MFS1* is modulated by regulatory elements upstream of the gene (7, 58). We confirmed that *MFS1* is likely contributing to DMI resistance through regulatory tuning by identifying 103 mapped k-mers upstream of the gene. These k-mers are likely capturing TE insertion variation modulating gene expression, such as the previously identified TE insertion 519 bp upstream associated with prochloraz, epoxiconazole, and prochloraz sensitivity shifts (7). Neither *Cyp51* nor *MFS1* was amenable to SNP-based GWAS discovery in previous studies on *Z. tritici,* and their implication in resistance was identified previously only through mutant analyses and crosses (7, 55). Mapping *Cyp51* and *MFS1* is consistent with the substantial increase in mapping power of the European diversity panel. We note, though, that we were unable to replicate an association of a DHHC palmitoyltransferase locus with propiconazole resistance (22). The geographic scope of the palmitoyltransferase association was restricted to a North American population, though, suggesting a lack of convergence on this mechanism in Europe. Finally, even though the ABC transporter gene *Atr1* and its promoter were shown to contribute to cyproconazole resistance in mutants (73), no genetic variants were found to be significantly associated. We cannot rule out the involvement of Atr1 overexpression in our panel, since one of the mapped transcription factors might indeed drive Atr1 expression, similar to how the transcription factor Mrr1 regulates the ABC transporter AtrB in *Botrytis cinerea* (8, 17, 74). Lack of association in ABC transporters or promoters thereof may also be due to differences in the genetic background of the strains, variation in experimental conditions to assess DMI resistance, or that the European diversity panel did not capture the relevant mutations at a high enough frequency (detection limit of 5% in SNPs).

In addition to the effective mapping of variations driving DMI resistance at known loci, we identified a broader set of previously undescribed resistance factors. These included seven genes encoding potential drug export functions by transporters belonging to the MFS beyond *MFS1*, and seven transcription factors. The associations in MFS genes were narrower across the spectrum of DMIs. This may be due to the variation in chemical structures among DMIs and possible links to specific fungicide-exporting capabilities by each MFS. If MFS transporters confer resistance to specific DMI subclasses, fungicide deployment might be optimized to account for previously unknown standing MFS-associated resistance in the field. Transcription factors modulating azole resistance have been supported by molecular characterizations in, e.g*., B. cinerea* and *Saccharomyces cerevisiae* (75, 76). We mapped four transcription factors associated with prochloraz resistance in colony growth conditions and three transcription factors associated with tebuconazole resistance based on EC_50_. In previous work, transcription factors were often indirectly linked to DMI resistance because they tend to regulate multiple genes, challenging the establishment of causal links with DMI resistance. The discovery of transcription factors associated with DMI resistance underpins the central role of transcriptional reprogramming in mediating fungicide resistance in very different fungal pathogens (72, 77). Our approach to combining multiple independent genotyping and phenotyping approaches has significantly expanded the scope of mapped DMI resistance variation. While the discovered gene functions provide plausible functional links to resistance, further targeted analyses are needed to clarify their specific roles. A systematic application of allele-swapping experiments would be necessary to assess the functional significance of the individual mutations discovered, e.g., in genes encoding efflux pumps and transcription factors.

### Emergence of resistant *Cyp51* haplotypes

The role of missense mutations in *Cyp51* has been extensively assessed experimentally; whether synonymous mutations can impact resistance remains unknown (78). Here, we discovered numerous significant associations of both synonymous and missense mutations in *Cyp51*. GWAS linked missense variants to previously functionally validated alleles. We found that Ile381Val and Ser524Thr were associated with prothioconazole and epoxiconazole resistance, respectively. We also found discrepancies in variants associated with prochloraz resistance. For instance, we found no evidence for an involvement of Ser524Thr even though previous mutant analyses demonstrated an effect (55). These discrepancies may be due to epistatic interactions among *Cyp51* mutations, where the combined effects of multiple mutations create unique resistance profiles that vary depending on the specific DMI tested. Epistatic effects of *Z. tritici Cyp51* mutations were demonstrated using functional replacement in yeast (55). The combination of Leu50Ser and Tyr461Ser missense mutations conferred an increase in resistance by a factor of six to epoxiconazole and a factor of 52 to tebuconazole. Adding the Ser524Thr mutation to the two previous mutations increases the resistance factor to 95 for epoxiconazole and 632 for tebuconazole. Furthermore, some mutations tested in isolation produced no functional Cyp51, highlighting co-dependencies of resistance-conferring mutations in both conferring resistance and maintaining enzymatic function.

Beyond epistatic effects of resistance-conferring mutations, GWAS are sensitive to LD in mapping populations. Strong recent selection of resistant *Cyp51* haplotypes is expected to increase the association of individual mutations at short physical distances. Combining GWAS discovery with specific genetic validation of associated haplotypes will alleviate these constraints on mapping power. In this study, we tested whether synonymous *Cyp51* mutations that accumulated during resistance gains on the European continent could contribute to resistance. Synonymous mutations have emerged recently as relevant targets to assess hidden genetic factors underpinning trait variation. Synonymous mutations can affect, among others, gene expression via mRNA stability or translation efficiency. Here, we introduced either the full complement of gained synonymous and missense *Cyp51* mutations into a sensitive background or only missense mutations. The full exchange for a resistant *Cyp51* haplotype significantly increased resistance, consistent with previous findings; however, no significant contribution of synonymous mutations was detectable, suggesting these associations were due to linkage disequilibrium. Our gene-swapping approach can be more broadly applied to reveal epistasis and decipher further complex genetic contributions in the most polymorphic loci, including assessing genetic factors in untranslated regions (UTRs), as well as upstream and downstream regions influencing gene expression. Additional experimental work will indeed be required to clarify individual locus contributions in regions with high degrees of linkage disequilibrium and potential epistasis such as the region surrounding *Cyp51*.

In conclusion, our work establishes a highly diverse panel of field-collected strains of a crop pathogen capturing gains in DMI resistance at the level of the European continent. The large body of associated variants across diverse gene functions highlights the complexity of resistance gains in agriculture. Managing resistance remains a challenge for agricultural production, but genomic monitoring opens powerful perspectives on this process. Development of new compounds may benefit from understanding the genetic mechanisms underlying resistance and facilitating the targeting of novel pathways. Additionally, integrating field-specific resistance surveillance with predictive genomic tools will enable more effective and sustainable fungicide deployment strategies.

## MATERIALS AND METHODS

### European diversity panel

For resistance monitoring purposes, Syngenta sampled pathogens from commercial and trial sites across Europe. The fungicide resistance monitoring efforts for *Z. tritici* led to a collection of 8,607 strains. From this collection, we defined a representative subset to conduct this study on DMI resistance. Strains were selected from the collection based on a hierarchical clustering approach grouping sampling locations into 100 km radius areas. Within each area, we balanced the collection over the 2005–2019 sampling years based on availability. The assayed European diversity panel included 1,394 strains from 27 different countries. A total of 286 strains were previously sequenced (29) (see Table S1 at https://doi.org/10.5281/zenodo.17063534), and 1,134 were sequenced for this study. The European diversity panel was contrasted against an additional 736 strains from a global diversity survey of the pathogen (31) (see Table S1 at https://doi.org/10.5281/zenodo.17063534).

### Purification of the European diversity panel

All strains included in the European diversity panel were collected from infected leaves across Europe, preferably with visible pycnidia, wrapped in dry paper towels, and packed into a paper envelope for shipment. Upon arrival in the laboratory, samples were labeled with a unique code, and the arrival date was recorded. The leaves were dried, wrapped in fresh paper towels if necessary, and stored at 4°C until further processing. Leaves with symptoms were cut into 2 cm pieces, surface-sterilized in 2% bleach for 2 minutes, and rinsed with sterile distilled water. Leaf cuts were placed on wet filter paper in Petri dishes (1.3 mL water for 9 cm dishes) and incubated at 20°C for 24 hours. Single strains were picked from cirrhi under a binocular microscope and transferred to V8 agar plates with antibiotics. Plates were incubated for 4–7 days at 20°C. To ensure that only a single genotype was collected per sample, a single spore isolation step was performed for every sample. Single colonies were subcultured on fresh V8 plates and incubated under the same conditions for an additional 7 days. Each strain was stored independently in a cryovial preserved in liquid nitrogen. Fresh cells harvested from V8 plates incubated at 18°C for a period of 5 days were used as inoculum for all experiments. Media recipes used in this study are summarized in Table S8, available at https://doi.org/10.5281/zenodo.17063534.

### Fungicide sensitivity assays

Microtiter plate liquid fungicide resistance assays were conducted to determine the EC_50_ as follows. Spore suspensions were obtained from yeast peptone dextrose (YPD) plates incubated for 6 days at 20°C in the dark and standardized to reach a density of 100,000 spores per milliliter using a Neubauer counting chamber. Fungicides, pre-dissolved in dimethyl sulfoxide (DMSO), were added at 1% vol to liquid yeast bacto glycerol (YBG) medium carrying the spore suspension (100 µL) (79). For prothioconazole and cyproconazole, tested concentrations were obtained through a fourfold cascade dilution series, starting from 40 mg L⁻¹ and sequentially decreasing to 0.0098 mg L^−1^ (seven inhibitor concentrations + DMSO control), for prochloraz, epoxiconazole, and tebuconazole starting from 10 and sequentially decreasing to 0.0024 mg L^−1^ (seven inhibitor concentrations + DMSO control). Sterile 96-well microtiter plates with lids (Costar) were incubated for 6 days at 20°C in the dark, and measurements were performed at 405 nm absorbance using a plate reader (EnVision 2105, PerkinElmer, Waltham, USA). EC_50_ values were calculated using AGSTAT, developed by Syngenta, using a non-linear curve fit. EC_50_ values were assessed across two technical replicates and two biological replicates. Specifically, biological material was prepared twice independently, followed by inoculation of two independent dilution series from each material. Details on the mean values for all tested fungicides can be found in Table S1 (https://doi.org/10.5281/zenodo.17063534). Comparison of prothioconazole EC_50_ values (sampling years 2016–2018) across strains from Germany, the UK, France, Ireland, and other European regions was tested using an ANOVA and *post hoc* Tukey’s honestly significant difference (HSD) tests.

Fungicide sensitivity on solid medium was determined using colony size estimates (fixed fungicide concentration). The European diversity panel strains stored at −80°C were arrayed in 96-well cryo-stock plates in sterile solution carrying 8.5% skim milk (m/vol) with 10% glycerol (used as mother array plates). From the mother array plates, the panel of strains to be tested was transferred using a 96-floating pin replicator tool (408FS2AS, V&P Scientific Inc.) to 96-well flat-bottom plates (model 3370, Corning) pre-filled with 100 µL YPD liquid medium and left to grow at 18°C for 7 days to reach a growth plateau. Spore suspensions were not further standardized and directly spotted using a Rotor HDA (Rotor+) (Singer Inc., Watchet, UK) with sterile pins (RePads) (Singer Inc., Watchet, UK) onto glycerol yeast extract (GlYE) agar media plates (PlusPlates) amended or not with fungicides. The fungicides were dissolved in DMSO and then mixed with GlYE agar to achieve a final concentration of 1% DMSO. Five different fungicide concentrations were applied to achieve final concentrations of 50, 10, 1, 0.1, and 0.01 mg L^−1^ per plate. Single concentrations were then selected for further GWAS. Control plates only contained GlYE agar with 1% DMSO. Spotted GlYE agar plates were incubated at 20°C for 7 days in the dark before imaging. Image capture was performed using a Phenobooth (Singer, Watchet, UK), and image analysis was performed with the Phenosuite (version 2.21) software package (Singer Inc., Watchet) by comparing paired growth areas of colonies grown on control plates without fungicide (DMSO controls) with fungicide-amended plates. The relative growth area on amended media (area_e_) relative to controls (area_c_) was estimated using the following formula:


$$Relative\;growth=\frac{area_{e}-area_{c}}{area_{c}}\times 100$$

Plates with signs of contamination were excluded. Plates with >2× more growth on fungicide-amended plates vs controls were excluded as well. Relative growth was estimated using single biological replicates, and assayed fungicides are reported in Tables S1 and S9, available at https://doi.org/10.5281/zenodo.17063534. We transformed relative colony growth values to a binary growth/no-growth trait reflecting resistant versus susceptible genotypes. To convert the quantitative data into a binary format, we assigned a value of 1 to all strains with relative growth greater than 0 and 0 to susceptible strains. The resulting binary data set is provided in Table S1 (at https://doi.org/10.5281/zenodo.17063534) in the columns “Binary_”.

Fungicide sensitivity of the transformants was determined in liquid culture assays. Sensitivity assays were performed as previously described with the following differences: spore suspensions were obtained from YPD plates rather than YBG, with identical incubation steps. For epoxiconazole, mefentrifluconazole, and benzovindiflupyr (SDHI control), 11 concentrations and a DMSO control were tested for each fungicide. The assayed concentrations were obtained through a fourfold cascade dilution series, starting from 40 mg L⁻¹ and sequentially decreasing to 0.000038 mg L^−1^. Growth measurements were performed at 600 nm absorbance using a plate reader (EnVision 2105, PerkinElmer, Waltham, USA). EC_50_ values were calculated using four-parameter non-linear curve fitting using the software GraphPad Prism 10. To assess the effect of *Cyp51* repression, 30 mg L^−1^ of doxycycline was added to the sensitivity assays.

### DNA extraction, Illumina sequencing, and SNP calling

Whole-genome sequencing data were produced from high-quality genomic DNA extracted using the DNeasy Plant Mini Kits (Qiagen Inc.) following the manufacturer’s instructions. Paired-end sequencing of 250 cycles with an ~500 bp insert size was performed by Novogene Inc. using the Illumina NovaSeq 6000 platform. We used Trimmomatic v.0.39 to trim low-quality sequencing reads and remove adapter contamination in each strain (80). Filtered sequences were aligned to the *Z. tritici* reference genome IPO323 (39, 81) using Bowtie2 v.2.3.3 (82). The Genome Analysis Toolkit (GATK) v.4.0.1.2 (83) was used for SNP calling and variant filtration. The GATK HaplotypeCaller was run with the command -emitRefConfidence GVCF and -sample_ploidy 1. Joint variant calling was performed using the tool GenotypeGVCFs, merging HaplotypeCaller gvcfs produced for an additional 1,022 from a previous study (29) with the option -maxAlt 2.

### Population genetic analysis and linkage disequilibrium

The joint genotyping calls for the European diversity panel (*n* = 1,420 strains before filtering), supplemented by strains from additional continents (total *n* = 2,156 strains), were filtered using VCFtools v.0.1.16 (84). For population structure analyses, we thinned the SNP set to retain variants satisfying the following criteria: a single variant per 1,000 bp (--thin 1000), a minor allele frequency (MAF) ≥0.05 (--maf 0.05), and less than 10% missing genotypes (--max-missing 0.9). This filtering retained 22,614 SNPs. We performed a principal component analysis using the read.vcfR and the dudi.pca functions in R (85). To analyze genetic structure within the European diversity panel, the same filtering was applied to the 1,420 strains, retaining 22,710 SNPs. To perform maximum likelihood estimation of individual ancestries, we used Admixture v.1.3.0 (86). We chose an admixture model independent of prior population information and with correlated allele frequencies. The admixture model was run with 10,000 iterations. We assessed K between 2 and 20, with five repetitions per K. The most likely number of populations (K) was estimated with the cross-validation method, and the output was analyzed with the R package Pophelper (87). The genetic distance (*D*_ST_) between individuals was estimated using PLINK v.1.90 (81). Finally, geographic distance between strains was calculated using the distHaversine function of the geosphere R package (88) (see Table S10 at https://doi.org/10.5281/zenodo.17063534).

We analyzed the decay in LD on chromosome 1. For this, we used all SNPs with a minor allele frequency ≥5%. We calculated the linkage disequilibrium *r^2^* between marker pairs using the options --hap-r2 and --ld-window-bp 10000 in VCFtools v.0.1.15 (84). The decay of LD with physical distance was estimated using a non-linear regression model (89, 90).

### SNP-based genome-wide association mapping

For SNP-based GWAS on the European diversity panel, we retained SNPs with a genotyping call rate of ≥90% and a MAF ≥ 5%, resulting in a final set of 472,103 biallelic SNPs (see Table S1 at https://doi.org/10.5281/zenodo.17063534). Traits for GWAS included the EC_50_ estimates for tebuconazole, cyproconazole, prothioconazole, epoxiconazole, and prochloraz. For relative growth, we included the same compounds along with mefentrifluconazole (see Table S2 at https://doi.org/10.5281/zenodo.17063534). We accounted for relatedness by constructing a genetic relatedness matrix (GRM) among strains using all genome-wide SNPs with the option “-gk 2” in GEMMA (91). Thus, all the associations were performed using a univariate linear mixed model (MLM + K) where K is the GRM as a random effect:


$$y=Wa+x\beta +u+\varepsilon ;u\sim MVN_{n}\left(0,{\lambda \tau }^{-1}K\right),\varepsilon \sim {MVN}_{n}(0,\tau^{-1}I_{n})$$

*y* represents a vector of phenotypic values for *n* individuals; *W* is a matrix of covariates (fixed effects with a column vector of 1); *α* is a vector of the corresponding coefficients, including the intercept; *x* is a vector of the genotypes of the SNP marker, *β* is the effect size of the marker; *u* is a vector of random individual effects; *ε* is a vector of random error; *τ*^−1^ is the variance of the residual errors; *λ* is the ratio between the two variance components; *K* is the *n* × *n* GRM; *I_n_* is an *n* × *n* identity matrix; and *MVN_n_* represents the multivariate normal distribution. We applied a stringent Bonferroni threshold (*α* = 0.05; *P* = *α* / total number of SNPs) to identify the most robust SNP associations. Significant SNPs were annotated using snpEff v.5.0e (92). We provide all association mapping outcomes in Table S3 at https://doi.org/10.5281/zenodo.17063534.

### K-mer-based genome-wide association mapping

We performed k-mer-based GWAS on the same 11 traits used for SNP-based GWAS, following a previously described approach (93). We used k-mers of 25 bp length, as suggested for small genomes such as *Z. tritici,* following previous studies (32). Quality-filtered sequencing reads (see above) of 1,406 strains from the European diversity panel were used for k-mer screening. K-mers were counted using a two-step process: the canonization involved treating each k-mer and its reverse complement as equivalent. In contrast, non-canonization treats the k-mer and its reverse complement as distinct entities. A k-mer and the reverse complement are supposed to have the same chance to appear in fastq files. However, technical reasons can create a bias toward one of the forms for some k-mers. Therefore, k-mers were then filtered based on two criteria: (i) a k-mer must appear in both its canonized and non-canonized forms in at least 5% of the strains, and (ii) it must appear in both forms in at least 20% of the strains in which it was found.

A GRM was estimated with EMMA (Efficient Mixed-Model Association) as an IBS matrix under the assumption that each k-mer has a small, random effect on the phenotype. GWAS were performed by using a linear mixed models (LMM) + K model in GEMMA with a likelihood ratio test to determine *P*-values. Beta estimation and significance testing were performed using the patched version of kmers_gwas.py, incorporating the recommendations outlined (https://github.com/voichek/kmersGWAS/issues/53 and https://github.com/voichek/kmersGWAS/issues/91). A k-mer was significant when the *P*-value passed the permutation-based threshold as described by Voichek and Weigel (93). We attempted to map all significant k-mers for each trait to the reference genome using the short-read aligner Bowtie v.1.2.2 (82) with the command “-a --best --strata” retaining the k-mers with a unique alignment. We used the center position of the mapped k-mer on the reference genome as a coordinate to inspect nearby features using BEDtools v.2.31.0 (94). In total, the number of k-mers consisted of 1,159,029,650 unique k-mers. *Cyp51* sequences were used for the construction of an unrooted phylogenetic network with SplitsTree v.4.17.1 (95). We provide links to all significant association mapping outcomes in Table S3 at https://doi.org/10.5281/zenodo.17063534.

### Structural variation-based genome-wide association mapping

We identified insertion and deletion variants ≥30 bp in 18 reference-quality genomes (96) by aligning these to the IPO323 reference using MUM&Co (97) and minimap2 + paftools (98). We used Jasmine (99) to combine the structural variant predictions from each genome into a single variant catalog with 25,839 non-overlapping sites based on IPO323 reference genome coordinates. The variant catalog was used to build a pangenome graph with the vg toolkit (100). Graph variants were genotyped in all strains by mapping quality-trimmed Illumina reads to the graph using Giraffe (101). We set all genotype calls with fewer than four supporting reads to missing and excluded all multi-allelic and invariant sites using GATK v.4.2.41 (102). GWAS were performed on a subset of 1,135 strains (see Table S1 at https://doi.org/10.5281/zenodo.17063534) using the software GEMMA (91). We accounted for relatedness by including a GRM and filtered variants for MAF ≥0.05. We provide data on all significant association mapping outcomes in Table S3 at https://doi.org/10.5281/zenodo.17063534.

### Gene functions and heritability

Genes located near significant variants were identified based on the functional annotation of the IPO323 genome (66) and using the “intersect” command in BEDtools v.2.29.2 (94). Gene ontology (GO) terms for encoded proteins were determined using InterProScan v.5.36-75.0 with default parameters (103). GO enrichment analyses were performed using the R packages GSEABase v.1.35.0 and GOstats v.2.38.1 (104). GO enrichment analyses were performed only for GO terms with a minimum term size of five genes and a false discovery rate of 0.01 in hypergeometric tests. The proportion of phenotypic variance explained (PVE) by genotypes, often referred to as “chip heritability,” was estimated using the LMM implemented in the EMMA software (91) (see Table S11 at https://doi.org/10.5281/zenodo.17063534). This approach allows for the inclusion of both fixed and random effects, thereby accounting for population structure and relatedness among individuals, contributing to more robust estimates of heritability.

### Molecular methods for transformations

All plasmids used for transformation were derived from the previously described binary vector pNOV2114 (105). Inserts were designed *in silico* and synthesized by GENEWIZ Inc. (Germany). To functionally replace the genomic *Cyp51* IPO323 copy with doxycycline-repressible *Cyp51* haplotypes, we first generated a ΔKU70::TtA* strain carrying an expression cassette of the TtA* (106) at the KU70 locus (binary plasmid pNOV2114-Ku70_TetRep_NPT2_Ku70; Data S1). We then designed three plasmids to replace *Cyp51 in-locus*: (i) AltsdhC_Hygr_pNOV2114_CYP51_IPO323, representing the IPO323 haplotype, (ii) AltsdhC_Hygr_pNOV2114_CYP51_IRL021, representing the haplotype of strain 15IRL021 and (iii) AltsdhC_Hygr_pNOV2114_CYP51_IRL021_SYN, representing the haplotype of strain 15IRL021 but carrying all synonymous mutations matching the IPO323 haplotype (see Table S12 at https://doi.org/10.5281/zenodo.17063534). The inserts obtained by gene synthesis were cloned between *EcoRI* and *HindIII* sites. To ensure full gene replacement, two different resistance cassettes were used, placed upstream and downstream of *Cyp51*, respectively. The flanking region of 1,135 bp upstream of *Cyp51* is followed by an isofetamid resistance cassette (PtrpC::alt-sdhC::MoILV term in reverse orientation; Data S2), followed by a TetO promoter (106) driving tetracycline-repressible overexpression of *Cyp51* haplotypes. Downstream of the 3′ end of *Cyp51*, a hygromycin-resistant cassette was inserted, followed by 1,042 bp corresponding to downstream genomic sequence (see Fig. 5A for graphical overview, and Table S13 and Supplementary Data at https://doi.org/10.5281/zenodo.17063534). *Agrobacterium tumefaciens*-mediated transformations were carried out following established protocols (105) for *Cyp51 in situ* replacement. The IPO323DKu70::TtA background was selected on G418 (250 mg L^−1^ on GlYE agar media), and subsequent *Cyp51* swapping transformants were selected on combined hygromycin and isofetamid selection (in GlYE media, at concentrations of 100 mg L^−1^ and 10 mg L^−1^, respectively). Clones were confirmed by PCR using primer combinations and PCR conditions listed in Table S13 (https://doi.org/10.5281/zenodo.17063534).

## Data Availability

Genome sequencing data are available on the NCBI Sequence Read Archive (accession numbers are provided in Table S1 at https://doi.org/10.5281/zenodo.17063534).
